# Caudal Regression and Encephalocele: Rare Manifestations of Expanded Goldenhar Complex

**DOI:** 10.1155/2017/4396142

**Published:** 2017-09-12

**Authors:** Gabriella D'Angelo, Lucia Marseglia, Salvatore Aversa, Sara Manti, Caterina Cuppari, Mariaconcetta Cutrupi, Carmelo Salpietro, Eloisa Gitto

**Affiliations:** ^1^Neonatal and Pediatric Intensive Care Unit, Department of Human Pathology in Adult and Developmental Age “Gaetano Barresi”, University of Messina, Messina, Italy; ^2^Division of Neonatology, Spedali Civili, Brescia, Italy; ^3^Unit of Pediatric Genetics and Immunology, Department of Human Pathology in Adult and Developmental Age “Gaetano Barresi”, University of Messina, Messina, Italy

## Abstract

Oculoauriculovertebral spectrum, or Goldenhar Syndrome, is a condition characterized by variable degrees of uni- or bilateral involvement of craniofacial structures, ocular anomalies, and vertebral defects. Its expressivity is variable; therefore, the term “expanded Goldenhar complex” has been coined. The Goldenhar Syndrome usually involves anomalies in craniofacial structures, but it is known that nervous system anomalies, including encephalocele or caudal regression, may, rarely, occur in this condition. We report two rare cases of infants affected by Goldenhar Syndrome, associated with neural tube defects, specifically caudal regression syndrome and nasal encephaloceles, to underline the extremely complex and heterogeneous clinical features of this oculoauriculovertebral spectrum. These additional particular cases could increase the number of new variable spectrums to be included in the “expanded Goldenhar complex.”

## 1. Introduction

Oculoauriculovertebral spectrum (OAVS, OMIM 164210), also known as Goldenhar Syndrome (GS), is a complex condition characterized by abnormal prenatal development of facial structures derived from the first and second branchial arches of the embryo with a prevalence ranging from 1 to 9 : 100,000 [1]. It derives from a defect of blastogenesis involving primarily aural, oral, and mandibular development, resulting in microtia, mandibular hypoplasia, vertebral anomalies, and epibulbar dermoid/lipodermoids. Although these clinical features are pathognomonic of the syndrome, its expressivity is quite variable, with ~50% of patients being affected by other anomalies, including cardiac, renal, pulmonary, vertebral, and neurologic defects [1]. Therefore, the term “expanded Goldenhar complex” has been coined. Only few cases of association of GS and caudal regression syndrome (CRS) or nasal encephalocele have been reported, and the inclusion of these new features could expand the spectrum of the Goldenhar complex.

CRS is a congenital condition characterized as premature termination of the spinal column with or without associated soft tissue, osseous, or visceral anomalies. The occurrence of branchial, pulmonary, cardiovascular, gastrointestinal, urogenital, and skeletal malformations constitutes the axial mesodermal dysplasia complex (AMDC). In the past, it was suggested that the label “axial mesodermal dysplasia spectrum” could be used in rare cases of patients with features of both Goldenhar and CRS [2].

Encephalocele is a protrusion of the brain and/or meninges through a defect in the skull and can be numbered among neural tube defects (NTDs) [3]. It can be due to failure of neural tube closure, resulting in a bony defect through which herniation of neural tissue may occur. Based on the location, a commonly accepted system classifies encephaloceles as occipital encephalocele and encephalocele of the cranial vault, which represent about 80% of all encephaloceles, and frontoethmoidal encephalocele and basal encephaloceles, collectively known as nasal encephaloceles [3]. The incidence of encephaloceles in western countries is 1 in 35,000 to 40,000 births [4]. This rare entity has been documented only in few cases of children with GS [5].

We report two rare cases of newborns affected by GS, associated with neural tube defects, specifically CRS and nasal encephaloceles.

## 2. Case 1

**Figure 1:**
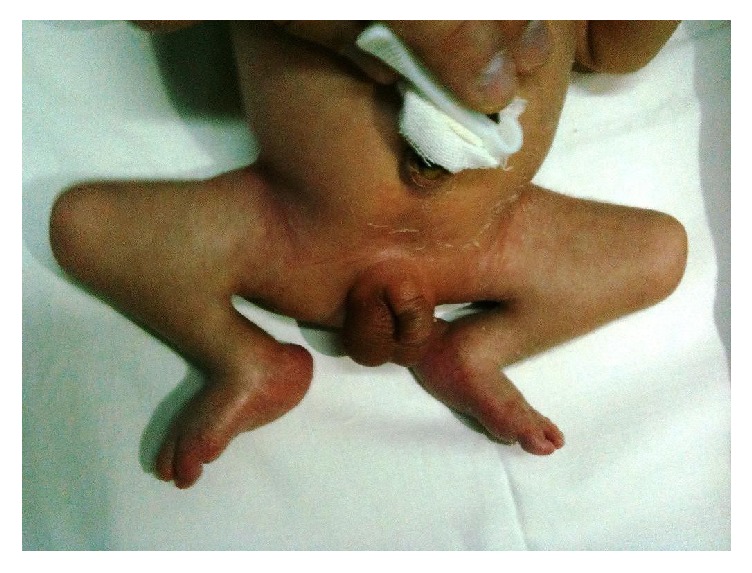
The “frog-like” appearance of the infant due to popliteal pterygium.

A male newborn was born by spontaneous vaginal delivery at 39 weeks of gestation to Indian nonconsanguineous parents. At birth, weight was 2220 g (−2SD), length 45 cm (−3SD), and head circumference 34 cm (−1SD). Apgar score was 9 and 10 at 1 and 5 minutes, respectively. Neonatal examination showed facial dysmorphic features, including marked facial asymmetry with right hemifacial microsomia, hypoplasia of the right mandibular ramus and condyle, bilateral blepharoptosis, and mild hypertelorism. Epibulbar and limbal dermoids, the ocular hallmarks of GS, were not present. Besides, the infant had severe hypoplasia of the right external ear with atresic external auditory canal and agenesia of ossicles. Choanal atresia was excluded and fiberoptic laryngoscopy did not show anomalies of the epiglottis and vocal cords. A “frog-like” appearance was noted in the lower extremities (Figure 1). His knees were flexed because of bilateral popliteal pterygium.

**Figure 2:**
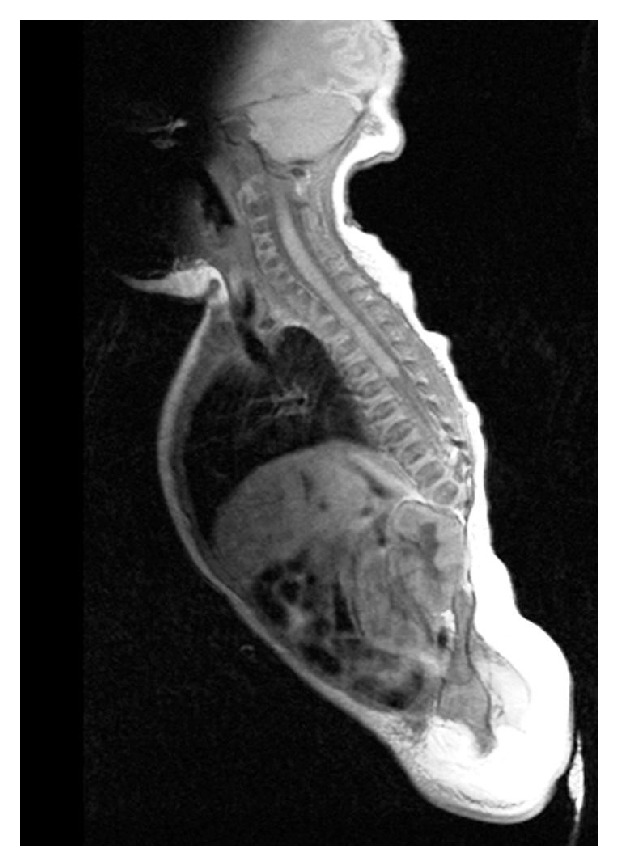
Sagittal T2W MRI of the spine shows severe caudal regression with complete absence of the lumbar spine and sacrum. The conus medullaris has a characteristic, abnormal, wedge-shaped (blunted) appearance.

Equinovarus deformity of bilateral feet was present. X-rays of the lower spine revealed absence of vertebra below D12 level. Spine magnetic resonance imaging (MRI) disclosed that the spinal cord terminated at approximately the D6 level (Figure 2). Lower limb somatosensory evoked potentials were normal; however, no signal was detected in the same district at electromyography. Brain MRI showed corpus callosum hypoplasia. Echocardiography revealed normally functioning bicuspid aorta. Ultrasonography of the abdomen detected crossed renal ectopia. A normal 46 XY karyotype was found.

## 3. Case 2

A 20-day-old Caucasian female infant was referred to our Neonatal Intensive Care Unit (NICU) for respiratory distress and poor feeding. She was born to a 32-year-old primiparous healthy mother, by normal spontaneous vaginal delivery at 38 weeks of gestation after an uneventful pregnancy. Prenatal history revealed abnormal ultrasonographic findings at the 24th week of gestation disclosing a single umbilical artery and, at the 34th week, cranial and facial asymmetry. Fetal karyotype was normal. Birth weight was 3320 gr, and Apgar score was 8 and 9 at 1 and 5 minutes, respectively. The infant's exam revealed an asymmetrical facial appearance with left hypoglossal nerve palsy, hypoplasia of the left mandibular ramus and condyle, and unilateral strabismus. Other abnormalities, such as lipodermoids, limbal dermoids, microphthalmia, cataract, and iris abnormalities, were not present.

**Figure 3:**
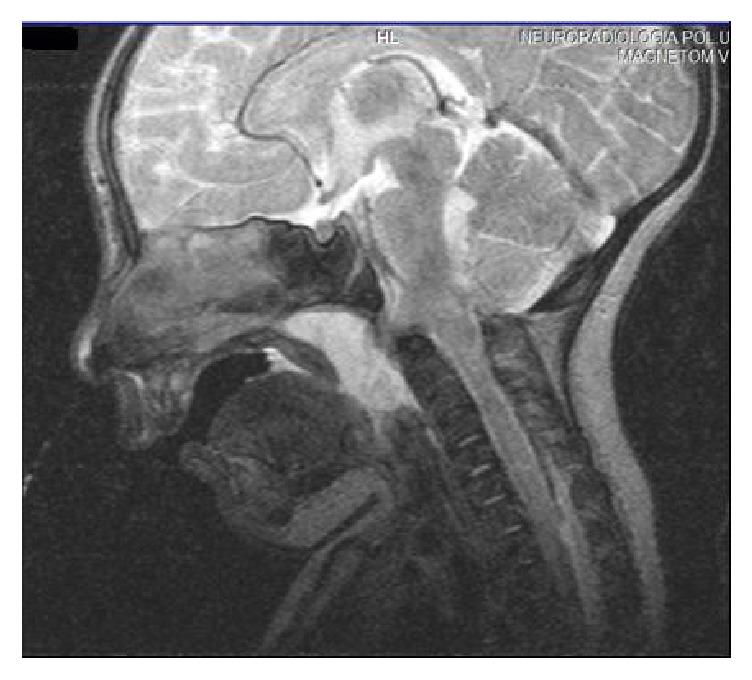
Sagittal T2W MRI of the skull shows a basal occipital meningoencephalocele with protrusion toward the naso- and oropharynx. The content of this malformation was primarily fluid with a small quantity of impacted bulb brain parenchyma.

As respiratory distress worsened, the infant required intubation, which was difficult due to the presence of a translucent mass in the pharynx. MRI revealed a basal occipital meningoencephalocele with protrusion toward the naso- and oropharynx. The content of this malformation was primarily fluid with a small quantity of impacted bulb brain parenchyma (Figure 3). CT imaging showed a suboccipital cranial cleft extending through a clival defect and a schisis of the anterior arch of the C-1 vertebral body and hypoplasia of the left mandibular ramus and condyle. X-rays of the higher spine revealed significant cervical scoliosis. Echocardiography was normal. For the surgical management of the meningoencephalocele, the infant was transferred to a pediatric neurosurgical center.

## 4. Discussion

GS or OAVS, first described in 1952 by Goldenhar [6], has been classified as a defect of blastogenesis, with time referred to all stages of development during the first 4 weeks of gestation. Most cases are sporadic, but various chromosome abnormalities have been associated with OAVS such as* trisomies 7, 8, 9, 10p*, and* 22* and deletions* 1p22.2–p31.1*,* 5p14*,* 18p*,* 22q11*, and* 22qter* [7]. It has been proposed that the presence of genetic variation, of variable penetrance and effect, combined with environmental factors, may affect specific tissue interactions that occur between cranial neural crest cells and the endoderm, mesoderm, and ectoderm, and the way they connect during migration in establishing the foundations of craniofacial morphogenesis may affect the risk of OAV [1]. Clinically, GS ranges from isolated microtia with or without mandibular hypoplasia, associated with microphthalmia, to a more complex phenotype with skeletal, cardiac, renal, pulmonary, and central nervous system manifestations [7]. In the literature, only few cases of GS with associated neuronal anomalies have been reported. In the cases described here, two rare associations of GS with CRS and encephalocele, both presumably resulting from failure of mesodermal cell migration during early blastogenesis, are reported. The patient described in case 1 presented with hemifacial microsomia, microtia, atresic external auditory canal, agenesia of ossicles, crossed renal ectopia, bicuspid aorta, bilateral popliteal pterygium, agenesis of vertebra below D12 level, and corpus callosum hypoplasia. Hemifacial microsomia and microtia are compatible with OAVS, while lumbosacrococcygeal agenesis is a manifestation of CRS. Typical manifestations of both GS and CRS are present in AMDC. CRS is characterized by symmetrical sacrococcygeal or lumbosacrococcygeal agenesis, most often accompanied by multiple musculoskeletal abnormalities of the pelvis and legs.

In 1981, Russel et al. labeled “axial mesodermal dysplasia spectrum” as a variety of combinations extending to different craniocaudal levels [2]. The patient described in case 1 is affected by axial mesodermal dysplasia, a rare complex of features of both Goldenhar and CRS. This patient presented a very serious clinical variant of AMDC. According to this theory, some abnormalities shown in our patient, such as CRS, might be explained as a midline anomaly secondary to genetic and/or environmental factors.

The patient described in case 2 presented with facial asymmetry, preauricular appendages on the left side, unilateral strabismus, and vertebral anomalies, considered to be cardinal findings of GS, associated with a nasal encephalocele. GS usually involves anomalies in craniofacial structures, but it is known that central nervous system anomalies, including encephalocele, only rarely occur in GS. There are multiple theories attempting to explain the formation of these basal encephaloceles [1, 4, 5], including the incomplete closure of the neural tube leading to herniation of meninges and neural tissue, and the persistence of the craniopharyngeal canal. Another theory points to failure of the neuroectoderm to separate from the surface of the ectoderm during formation of the neural tube, thus preventing mesodermal tissue from interposing between these two germ layers, causing alteration in skull ossification and allowing herniation secondarily. All the mentioned theories can explain the development of anomalies in our second case.

Basal encephaloceles, unlike frontoethmoidal encephaloceles, are not clinically visible due to their location within the nasal cavity and may present with upper-airway obstruction, as in our patient.

The true incidence of extrafacial anomalies expressed in patients with GS is not well known. In the expanded OAVS, extrafacial findings may be expressed to a greater degree. A summary of literature data shows occasional cases of coexisting encephalocele in patients with GS: Guzman [8] observed meningoencephalocele in at least ten cases; Gupta et al. [9] described a patient with an anterior encephalocele; Daisuke et al. [10] discussed the case of a neonate with GS and occipital meningoencephalocele, a cerebellocele, caused by dysraphism of the foramen magnum.

Therefore, it could be helpful to look for the presence of encephaloceles, whose prompt diagnosis modifies the care for patients.

In conclusion, we report two cases with particular associations to help obtain a more precise delineation of the phenotype of the “expanded Goldenhar spectrum,” underlining the notion that the extremely complex and heterogeneous clinical features may lead to a different and, sometimes hard, diagnostic definition.
